# Terahertz Spectroscopic Diagnosis of Myelin Deficit Brain in Mice and Rhesus Monkey with Chemometric Techniques

**DOI:** 10.1038/s41598-017-05554-z

**Published:** 2017-07-12

**Authors:** Yi Zou, Jiang Li, Yiyuan Cui, Peiren Tang, Lianghui Du, Tunan Chen, Kun Meng, Qiao Liu, Hua Feng, Jianheng Zhao, Mina Chen, Li-Guo Zhu

**Affiliations:** 1 Interdisciplinary Laboratory of Physics and Biomedicine, Institute of Fluid Physics, China Academy of Engineering Physics, Mianyang, Sichuan 621900 China; 2 Microsystem and Terahertz Research Center, China Academy of Engineering Physics, Mianyang, Sichuan 621900 China; 3 The State Key Laboratory of Biotherapy, West China Hospital, Sichuan University, Chengdu, 610041 China; 4 Department of Neurosurgery, Southwest Hospital, Third Military Medical University, Chongqing, 400038 China; 5 School of Electronic and Electrical Engineering University of Leeds, Woodhouse Lane, Leeds, LS2 9JT United Kingdom

## Abstract

While myelin deficit of the central nervous system leads to several severe diseases, the definitive diagnostic means are lacking. We proposed and performed terahertz time-domain spectroscopy (THz-TDS) combined with chemometric techniques to discriminate and evaluate the severity of myelin deficit in mouse and rhesus monkey brains. The THz refractive index and absorption coefficient of paraffin-embedded brain tissues from both normal and mutant dysmyelinating mice are shown. Principal component analysis of time-domain THz signal (PCA-tdTHz) and absorption-refractive index relation of THz spectrum identified myelin deficit without exogenous labeling or any pretreatment. Further, with the established PCA-tdTHz, we evaluated the severity of myelin deficit lesions in rhesus monkey brain induced by experimental autoimmune encephalomyelitis, which is the most-studied animal model of multiple sclerosis. The results well matched the pathological analysis, indicating that PCA-tdTHz is a quick, powerful, evolving tool for identification and evaluation myelin deficit in preclinical animals and potentially in para-clinical human biopsy.

## Introduction

In the central nervous system, oligodendrocytes wrap their branch-like extensions around axons to create myelin sheath, which is essential for the nervous system function^1^. Myelin damage-caused conduction failure results in paralysis, sensory-motor dysfunction, cognitive impairment, mental retardation or even death^2^. Diseases associated with myelin damage such as multiple sclerosis (MS), Charcot-Marie-Tooth disease, Pelizaeus-Marzbacher disease, schizophrenia, chronic depression etc.^2^ lead to neurological, even psychiatric disorders. A variety of methods including cerebrospinal fluid analysis, neuroimaging (e.g., MRI), serum testing and histological techniques of biopsy or autopsy proved effective in diagnosing myelin disorders^3–5^. However, in disorders with complex pathogenesis, such as MS, individual biomarkers in CSF or serum test are likely to reflect only isolated components of ongoing neuroinflammation and neurodegeneration, lacking predictive and prognostic significance^6^. Besides, the reliability correlates with the ‘severity’ of disease. In addition, there is a clear discrepancy between the clinical course of the disease and the ‘severity’ interpreted from the MRI results, also known as the clinico-radiological paradox^7^. The well-known methods for histological samples, such as confocal fluorescence and electron microscopy have been invaluable in furthering the cellular understanding of myelin development, plasticity and pathology^8^. However, these techniques need exogenous labeling and consume time. More efficient tools for identification and evaluation of the myelin damage-associated diseases need to be developed.

Recent studies show that terahertz (THz) spectroscopy, a state-of-the-art research field, is promising in applications in biology and medicine due to its unique features–label-free, non-invasive, non-ionizing, and high sensitivity to biomolecules and tissues^9–11^. THz spectroscopy has been applied to distinguish brain tumors^12–14^ and neurodegenerative disorders, such as Alzheimer’s disease^15, 16^, but it probably more apply to myelin-associated disease. In 2004, with continuous-wave THz imaging technique, Darmo *et al*. obtained images of a dehydrated rat brain section at 3.4 THz. Their images clearly identified the gray matter tissue (e.g., cerebral cortex) and the white matter tissue (e.g., corpus callosum) of the rat brain frontal sections. They concluded that the white matter has lots of myelin, which mainly consist of lipids, whereas, the gray matter of brain have a higher content of water and proteins, which gives higher signal intensities in THz images than the tissues with high lipid content^17^. Oh *et al*. obtained the reflection spectrums of gray and white matter in a range of frequencies from 0 to 2.5 THz and found the major difference was in 0.3 to 1.0 THz^18^. Using peak-to-peak values of the reflected pulse and bandwidths of 0.3–1.3 THz, they imaged a fresh rat brain and found that the absorbance and the reflectivity of terahertz waves of the white matter are lower than those of the gray matter^19^. The gray and white-matter regions were distinguishable by terahertz waves may owing to the different distribution of myelin in the brain tissue^19^. These results suggest that THz spectroscopy could detect myelin deficit brain.

In our study, THz absorption coefficient and refractive index of normal and mutant dysmyelinating mice^20, 21^ brain tissues were measured quantitatively. We distinguished the normal and the pathological tissues with high accuracy through either the relation between refractive index and absorption coefficient or principal component analysis of time-domain THz signal (PCA-tdTHz). With the brain tissues of a rhesus monkey model of experimental autoimmune encephalomyelitis (EAE), which is one of the fittest models for related research of MS disease^22, 23^, we demonstrated that PCA-tdTHz could be used for recognizing of demyelinated lesions and the results well matched the pathological analysis. The non-human primate EAE model bridges the gap between rodent models and patients, suggesting PCA-tdTHz as a new method for early clinical diagnosis of demyelinating diseases.

## Results

### Terahertz spectra of myelin deficit brain from *Rheb1* KO mice

**Figure 1:**
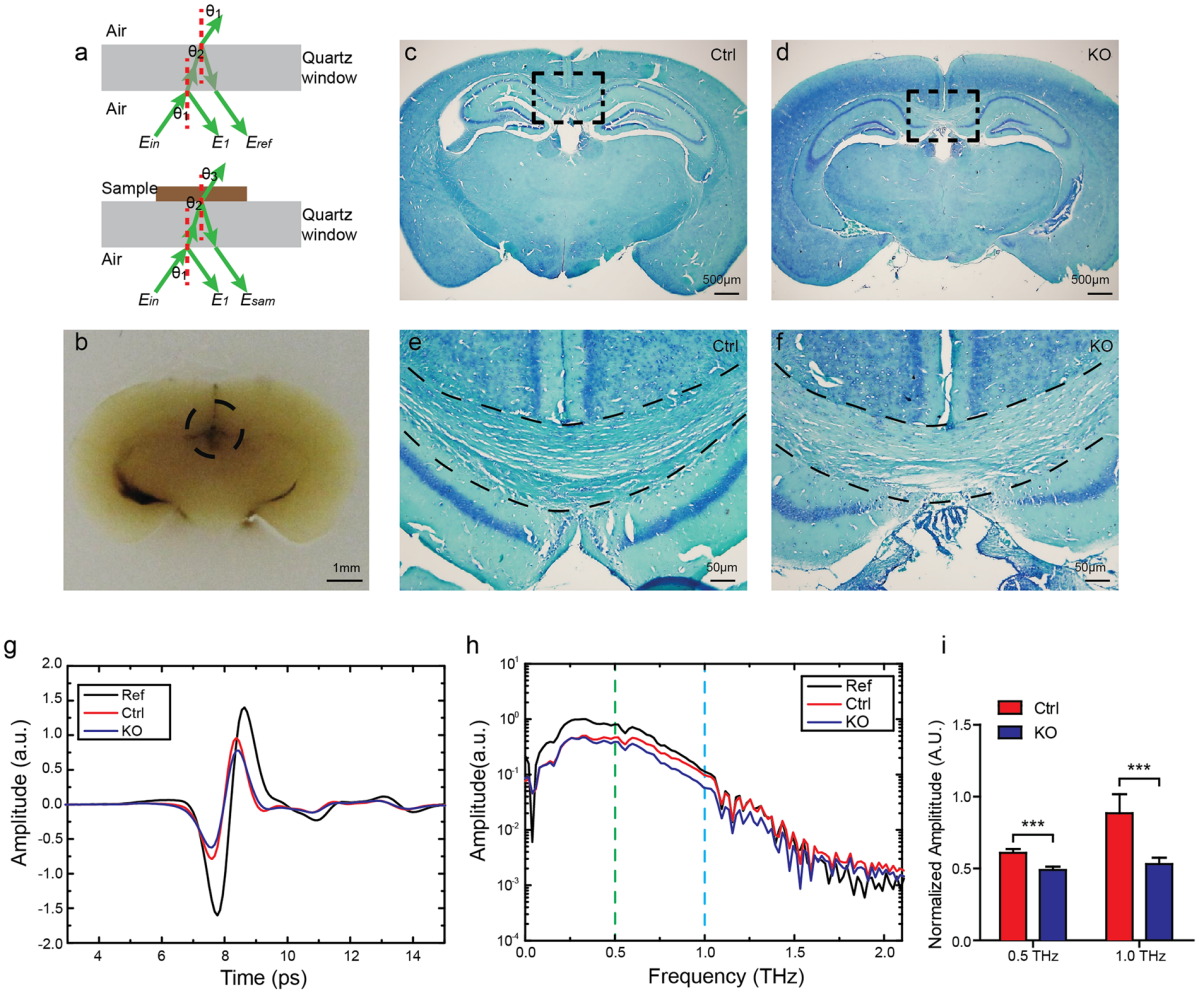
Reflected THz spectra of myelin deficit brain from *Rheb1* KO mice. (**a**) Schematic diagram of the reflective THz-TDS measurement. (**b**) White-field images of paraffin-embedded brain coronal sections from a mutant mouse. (**c**–**f**) Luxol-Fast Blue (LFB) staining shows myelin deficit in corpus callosum (regions in dash line) of *Rheb1* KO brain. (**g**) THz time-domain waveforms reflected by *Rheb1* KO brain samples (blue), normal brain samples (red) and the substrate without sample (black). (**h**) Fourier-transformed spectrum of the THz time-domain waveforms in (**g**). (**i**) Quantification of normalized amplitudes at 0.5 and 1.0 THz. Data represent mean ± SD, n = 3, ***p < 0.001.

We measured the myelin deficit and the normal mouse brain samples by a THz-TDS system in reflection mode (Fig. 1a, detailed measurement description can be found in Method Section). Brain samples from three mutant *Rheb1* ^*f/f*^; *Olig1* ^*Cre*+/−^ mice^21^, defined as *Rheb1* KO (Fig. 1b) demonstrated myelin deficit by luxol-fast blue (LFB) staining (Fig. 1c–f) and reductions of myelin genes in brain^19, 20^. The time-domain THz pulses of paraffin-embedded brain tissues were collected at ten measurement points for each sample. One of the points was in the center of the coronal section (denoted by black dashed circle in Fig. 1b), serving as the origin of an imaginary x-y plot, and the other nine points were equally spaced in ~250 μm from the origin on either x or y axis.

The average amplitudes of *Rheb1* KO and control brains were smaller than those of the reference pulse (Fig. 1g). However, the amplitudes of the average spectra of the myelin deficit brains were generally lower than those of the normal brains. Through a fast Fourier transformation of the time-domain signals, we obtained the frequency-domain spectra (Fig. 1h). Consistent with the time domain result, the amplitudes of the myelin deficit brains were lower than those of the normal brains in the frequency domain ranging from 0.3 to 1.0 THz. For quantitative analysis, spectrum amplitudes of *Rheb1* KO (n = 3) and control (n = 3) specimens were measured at 0.5 THz as previous described^14^ and 1.0 THz. These amplitudes were normalized to the amplitudes at 0.5 THz and 1.0 THz from each reference signals. The values of normalized amplitudes were 0.609 AU ± 0.027 (mean ± standard deviation [S.D.]) in control and 0.490 AU ± 0.023 in *Rheb1* KO (P < 0.001) at 0.5 THz (Fig. 1i). A significant difference was found at 1.0 THz with the value of 0.914 AU ± 0.084 in control and 0.530 AU ± 0.034 in *Rheb*1 KO (P < 0.001). The values in *Rheb1* KO were less than those in control, which may result from hypomyelination.

**Figure 2:**
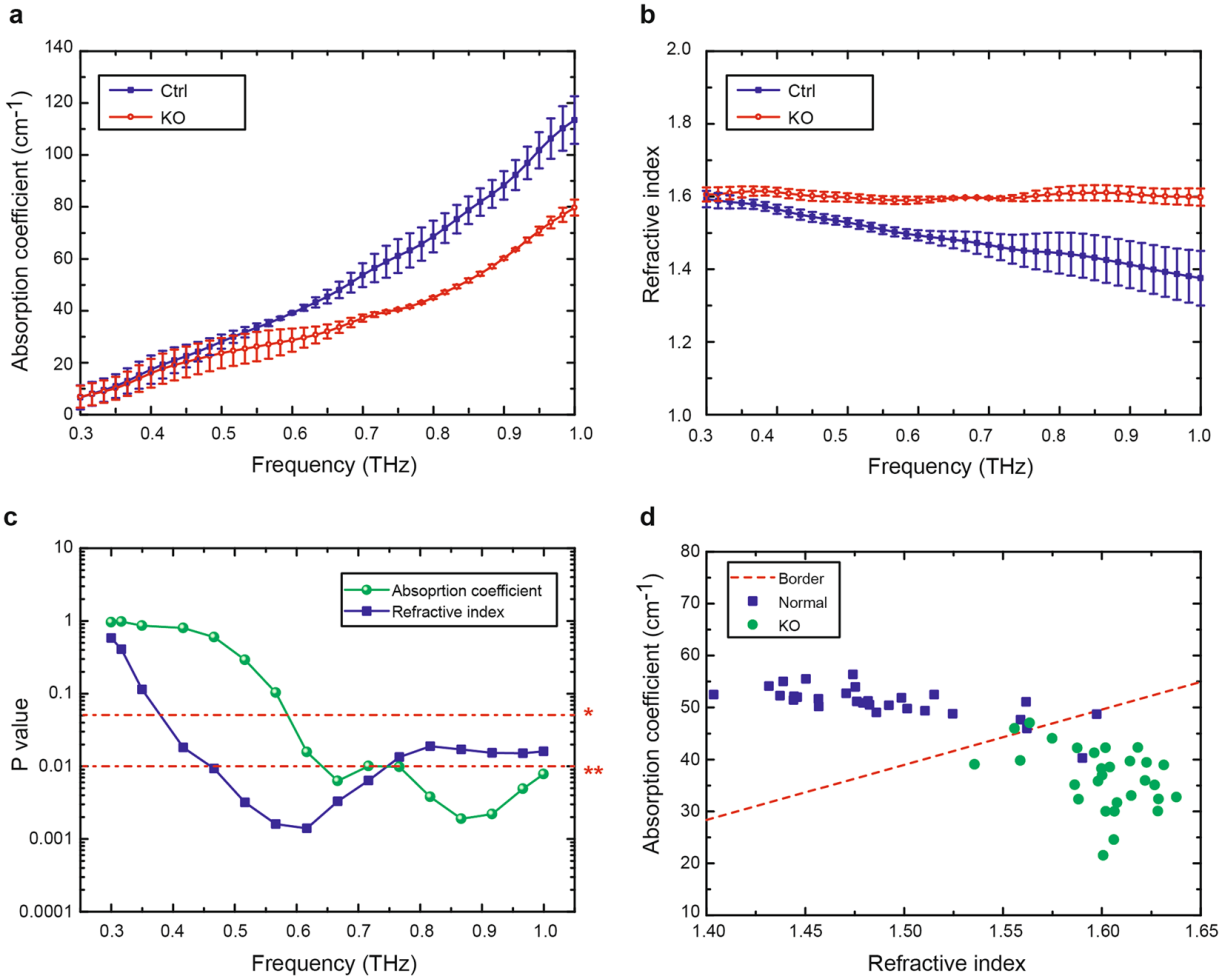
THz properties of myelin deficit brain from *Rheb1* KO mice in the frequency range from 0.3 to 1.0 THz. (**a**) Absorption coefficient of the paraffin-embedded brain samples from normal (blue solid square, Ctrl) and *Rheb1* KO (red empty circle, KO). (**b**) Refractive index of paraffin-embedded brain samples from normal and *Rheb1* KO. (**c**) P-value of absorption coefficient (green circular dot) and refractive index (blue square) results. *p < 0.05, **p < 0.01. (**d**) Score plot of the values for refractive index and absorption coefficient obtain from *Rheb1* KO (green circular dots) and normal (blue square) brains.

The absorption and the refractive index spectra were calculated (Fig. 2) by averaging over the obtained data of three *Rheb1* KOs and three control mice at ten measurement points. The S.D. were shown as error bars. Steady increasing of the absorption coefficients for brains from both groups were observed when the frequency increased from 0.3 to 1.0 THz. However, only a moderate increase in the absorption was observed in myelin deficit brain samples, in contrast to a significant increase in that of normal brain samples (Fig. 2a). Consistently, the refractive indices of the myelin deficit brains maintained at about 1.60 over a wide THz range from 0.3 to 1.0 THz, while those of the normal brains displayed a gradual decrease from 1.58 to 1.38 (Fig. 2b). We compared the absorption coefficients and refractive indices of *Rheb1* KO and control at different THz frequencies with Student’s t-distribution test at a significance level of 5% (P value). Statistical differences between *Rheb1* KO and control of absorption and refraction were from 0.6 to 1.0 THz and 0.4 to 1.0 THz respectively (Fig. 2c *p < 0.05, **p < 0.01).

To estimate the reliability of discrimination using the THz spectra, score plots of the refractive index and absorption coefficient values were made from the spectral data of all the measurement points (Fig. 2d). The value of a single point in the score plot was the average of the frequencies ranging from 0.3 to 1.0 THz. Using the linear discriminant analysis, a boundary line was described for distinguishing *Rheb1* KO and control brains (dashed line in Fig. 2d). A considerable discrimination accuracy of 93.3% was calculated by the ratio of the proportion of the correctly classified points to the total number of points. As a result, the range of frequencies, 0.3 to 1.0 THz, which reflects pathological changes of brain tumor^12^ and major difference between gray matter and white matter in rat brain^18^, shows good stability and repeatability in our experiment.

### Chemometric analysis of myelin deficit brain from *Rheb1* KO mice

**Figure 3:**
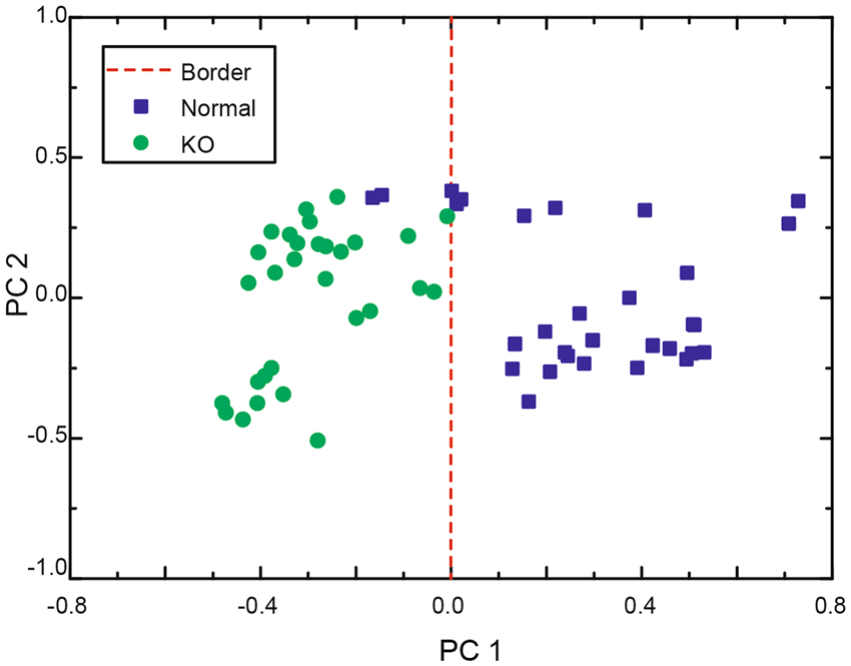
Chemometric analysis of myelin deficit brain and normal brain. Score plot of the first two principle components for the *Rheb1* KO (green circular dots) and normal mouse (blue square) brains samples. Red dashed lines show the boundary between the *Rheb1* KO and normal brain samples.

Brain is a complex system contains enormous molecules and unclear relationships. Proper experimental design, unsupervised data exploration and data mining techniques are required to identify these unclear relationships in a complex database. Chemometrics is a multivariate analysis field using statistics to compute models for highlighting the chemical differences and reducing variation due to physical effects. Principal component analysis (PCA) is one of the fundamental methods of chemometrics, which reduces the number of variations in data set to few principal components (PCs). PCA has been efficiently used in NMR and Raman spectra^24, 25^, and currently been applied to distinguish the genetically modified organisms (GMOs)^26, 27^ and even brain tumors in THz spectroscopy^13^. Thus, we performed PCA-tdTHz to discriminate the *Rheb1* KO and control mice samples. A score plot of the time-domain THz signals of the 6 mice brain samples was shown in Fig. 3. The first two PCs contained the most spectral variations of 85%. Similar to the statistical analysis based on the THz complex index parameters (Fig. 2d) we separated the myelin deficit brains from the normal brains according to PCA-tdTHz with a higher discrimination accuracy of 96.7% (Fig. 3). Without calculating the complex refractive index, PCA-tdTHz showed an advantage of time-saving and adapted better to examine the biopsy of patients with demyelinating neuropathies.

### PCA-tdTHz identified EAE lesions in non-human primate

**Figure 4:**
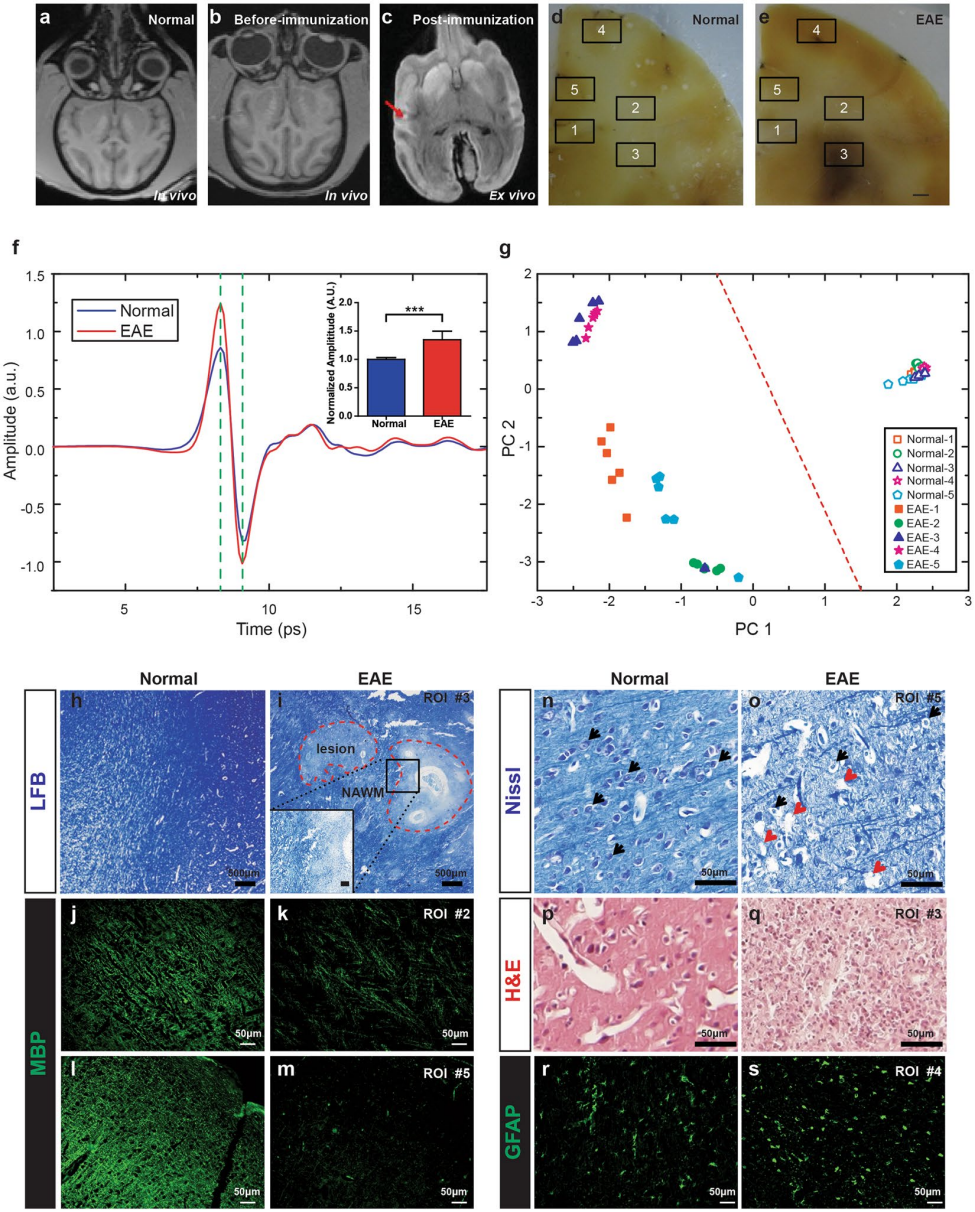
Distinguishing brain from EAE monkey by THz-TDS with PCA. (**a**–**c**) Examples show MRI of normal monkey, EAE monkey before immunization, and EAE monkey post-immunization. (**d** and **e**) White-field images of paraffin-embedded brain coronal sections from normal and EAE monkeys. (**f**) THz time-domain waveforms reflected from the regions of interest (ROIs) in normal monkey (blue) and EAE monkey brain samples (red). (**g**) Score plot of the first two principle components for the EAE and normal monkey brain tissues. The solid and hollow figures represent the calculation results for the EAE and the normal brains, respectively. (**h**,**i**) LFB staining shows completely demyelinated lesions with a sharp border (dashed line) and the surrounding normal appearing white matter (NAWM) in ROI #3 of EAE compare with normal white matter in control monkey. (**j** to **m**) Immunostaining with MBP antibody shows reduction of MBP+ fibers in ROIs #2 and #5 of EAE brain. (**n** and **o**) Dying neurons (arrows) with condensed nuclei and cavity caused by loss of neuron (arrowheads) were observed in ROI #5 of EAE brain. (**p** and **q**) Numerous foamy macrophages/microglia were infiltrated in ROI #3 of EAE brain. (**r** and **s**) Immunostaining of GFAP+ astrocytes in ROI #4 of EAE and normal monkey.

Since PCA-tdTHz excellently differentiated myelin deficit brains from normal brains in mice, we further tested this method in preclinical animal models of demyelinating diseases, such as MS. The EAE is one of the most characterized animal models of MS^23^ and EAE non-human primate model is essential for preclinical research and assessment^15^. EAE monkey was induced by immunizing with recombinant human myelin oligodendrocyte glycoprotein peptide (rhMOG_1–125_)^28^ (Fig. 4b). We measured paraffin-embedded brain tissues from both EAE and normal rhesus monkey with THz-TDS system as mentioned before. Compared with the control monkey (Fig. 4a), the EAE monkey showed paralysis associated with brain demyelination and hydrocephalus lesions indicated by postmortem MRI (arrow in Fig. 4c). We prepared coronal sections of EAE brain with the lesions identified by the postmortem MRI result, and the corresponding sections of the normal brain. For obtaining the time-domain THz signals of EAE and normal brain tissues, we randomly measured five comparable regions of interest (ROIs) in EAE and normal brain sections (Fig. 4d,e). Notably, ROI #3 with a large hemorrhagic/necrotic lesion in the EAE brain was visualized by naked eye (Fig. 4e). We positioned the THz wave focus (the origin of an imaginary x-y plot) close to the center of ROI. Another five points were equally spaced in 500 μm from the origin on either x or y axis. Generally, the average amplitudes of ROIs in EAE brain were higher than those in normal brain (Fig. 4f). We calculated the peak-to-peak values of the THz pulses and normalized them to the average value of normal monkey brain. The normalized amplitudes of EAE monkey were significantly increased (insert of Fig. 4f). However, PCA-tdTHz discriminated the EAE brain from the normal brain more clearly as shown in Fig. 4g. All ROIs in the normal brain were grouped on the right side with almost the same values of positive PC1 and nearly zero PC2, while ROIs in the EAE brain were on the left side with negative PC1 and diverse values of PC2. Unexpectedly, the different ROIs were separated in EAE by PCA-tdTHz. The score points of ROI #3 and #4 clustered together with the maximum negative PC1 and the maximum positive PC2 values, while the score points of ROI #2 were isolated by the near-zero PC1 and the maximum negative PC2 values. The score points of ROI #1 and #5 with similar negative PC2 values were separated by different PC1 values.

The EAE monkey showed completely demyelinated lesions (insert of Fig. 4i) in ROI #3 and severe hypomyelination in other ROIs of the brain indicated by LFB staining and immunostaining with myelin basic protein (MBP) antibody (Fig. 4j to m). In addition to myelin deficit, we observed concurrent neuronal death and inflammation in some ROIs of EAE brain. Compared with normal brain, numerous dying neurons (arrows) with condensed nuclei and extensive destruction of cavity (arrowheads) caused by massive loss of neurons were observed in ROIs #1 and #5 of EAE (Fig. 4n and o). Inflammation was observed in ROIs #3 and #4 infiltrated by numerous foamy macrophages/microglia (Fig. 4p and q) and reactive astrocytes (GFAP+, Fig. 4r and s). The pathological results showed that there were cellular and structural changes not only in lesions but also in normal appearing gray matter (NAGM) and normal appearing white matter (NAWM), which were normal appearing in MRI. Comparing the results of PCA-tdTHz and histopathology, different ROIs with different pathological subtypes clearly clustered among the two dimensions, suggesting unclear relationships between the PCA-tdTHz results and pathological damages.

## Discussion

This study proposed a novel approach based on THz-TDS combined with chemometrics to discriminate myelin deficit brain. Without exogenous labeling, PCA-tdTHz quickly distinguished the myelin deficit brain in both *Rheb1* KO mice and the EAE model of rhesus monkey. Although the hypothesis that THz spectroscopy would be applied to diagnosis of the demyelination diseases has been proposed during the past decade^17, 19^. To our knowledge, there is no direct experimental result to support this notion so far. We directly demonstrated the feasibility of THz spectroscopy in samples from transgenic mice and non-human primate EAE model–two models commonly used to study the neuropathological mechanisms and test the new therapeutic approaches of myelin-associated diseases such as MS^29^. Thus, this is a time-saving and label-free approach with great potential in preclinical studies of myelin pathologies.

Although the precise mechanism for the THz spectral difference between the myelin deficit and normal brain tissues is not clear, it probably relates to the cell density/category and the composition of myelin. First, remyelination, inflammation, reactive gliosis and axonal degeneration occur concurrently with myelin degeneration. Remyelination in the adult brain requires oligodendrocyte progenitor cells (OPCs), which are present in the white matter and gray matter of healthy brain as well as in multiple sclerosis lesions^30^. They respond locally to demyelination by proliferating and generating oligodendrocytes during remyelination^31, 32^. The interspersed viable neural tissue in the many overlapping perilesion perimeter regions is exposed to intense hypertrophic reactive gliosis and inflammation^33^. Demyelinated axons tend to atrophic and may eventually degenerate^34^. All these changes in cellular level would have direct impacts on THz spectrum, as suggested by the PCA-tdTHz result combined with histology of the EAE model in rhesus monkey. Second, the dry mass of myelin is characterized by a high proportion of lipid (about 70%) and a low proportion of protein^35^. We found that myelin deficit brains from *Rheb1* KO mice absorbed less THz wave. This is consistent with the hypothesis that loss of lipids in demyelinating disorders would be highlighted in THz imaging because of less absorption^17^. However, it is not appropriate to conclude that any distinction between the myelin deficit and normal brains is due to a specific type of component (e.g. lipid), since myelin proteins also reduced about 50% in *Rheb1* KO^20^. It would be necessary to investigate the mechanism for the THz spectral difference of the myelin deficit and normal brains in both cellular and molecular levels in the future.

Noteworthy, PCA-tdTHz effectively determined EAE and normal monkey brains, as well as the pathological changes in EAE such as demyelination (all ROIs), inflammation (ROIs 3&4) and neuronal death (ROIs 1&5), suggesting the potential of PCA-tdTHz to reflect and characterize the pathogenic processes. However, it is difficult to clarify the relationships between pathological subtypes and PCA-tdTHz results in EAE model since it is a complex model and do not accurately reflect the pathology of a progressive condition. Other animal models with specific pathologies or different pathological changes with time course would be useful. Biopsies and autopsies from patients with different demyelinating diseases, such as MS and PMD are also helpful to investigate the relationships between pathological subtypes and PCA-tdTHz results.

Compared with the conventional histological methods such as electronic microscopy (EM) and confocal microscopy, THz spectroscopy provides intrinsic optical property of normal and diseased tissue without labeling, thus faster and more stable. THz spectroscopy allows the investigation of underlying pathology when biopsy or autopsy is possible, which is limited in MRI due to both substantially lower resolution and relative non-specificity of intensity changes. As a state-of-the-art technology, THz spectroscopy may create a bridge between MRI and histology.

Results from a histological study of skin biopsies from patients with Charcot-Marie-Tooth disease suggest that skin biopsy may in certain circumstances replace the more invasive nerve biopsy in the morphological and molecular evaluation of demyelinating neuropathies^5^. This affords an opportunity for employing THz spectroscopy on clinical demyelinating disease discrimination, since THz spectroscopy has been used for studying human skin *in vivo* ^36^. Furthermore, mathematical models have shown that *in vivo* THz spectroscopy of the brain is plausible at higher power levels of THz source^15, 37, 38^.

In summary, we demonstrated the differences of the optical properties between myelin deficit and normal brains in THz frequency range. Our study on both transgenic mouse model and EAE monkey model indicates the PCA-tdTHz application in preclinical animal studies of myelin pathologies. This technique provides new opportunities for diagnosis of demyelination in biopsy specimens of human.

## Methods

### Brain tissue preparation

The myelin deficit mouse models for our study were generated as mentioned previously^20, 21^. Three 6-week-old knockout mice (*Rheb1* ^*f/f*^; *Olig1* ^*Cre*+/−^) and three control littermates (*Rheb1* ^*f/+*^; *Olig1* ^*Cre*+/−^ or *Rheb1* ^+/+^; *Olig1* ^*Cre*+/−^) weighing 22 ± 2 g and belonging to the C57BL/6 strain were used in our study. Recombinant human MOG 1–125 (rhMOG) are homemade. EAE rhesus monkey under ketamine anaesthesia was injected with a total of 700 μl of a 1:1 emulsion composed of 350 μg MOG1–125 peptide in PBS and complete Freunds’ adjuvant (CFA; Difo Laboratories; Detroit MI) as described previously^28^. The 700 μl emulsion was injected into the inguinal, axillary and regions of the dorsal skin. Monkey received 500 ng pertussis toxins (PTX) on days 0 and 2 after immunization. Monkeys were assigned clinical scores daily from 0 to 5. The care of laboratory animal and the animal experimental operation have been conforming to China Administration Rule of Laboratory Animal. All animal experimental protocols and euthanasia were reviewed and approved by Laboratory Animal Welfare and Ethics Committee of the Third Military Medical University.

The brains of mice and monkeys were collected and fixed in 10% formalin for 24 hr to 72 hr. After washed by current water, the whole formalin fixed brain were dehydrated, vitrified and infiltrated as described previously^8^. The paraffin-embedded tissues were cut into 3–5 μm coronal sections with Ultra-Thin Semiautomatic Microtome (Leica) for histology and the rest for THz spectroscopy measurement.

### Post mortem MRI and histology

T2-weighted MR images were recorded from formalin-fixed brains using the methodology described for the common marmoset EAE model^39^. The extent of inflammation, demyelination and axonal pathology were evaluated on tissue sections stained with hematoxylin/eosin (H&E) to visualize infiltrated cells; Luxol Fast Blue combined with Nissl for detection of myelin and neurons. MBP and GFAP antibodies for visualization of MBP+ myelinated fibers and GFAP+ reactive astrocytes were purchased from Calbiochem (San Diego, CA).

### THz-TDS measurement

To measure the THz signal, we used a homemade THz time-domain spectroscopy (THz-TDS) system in reflection mode (THz incidence angle 35°) with InGaAs photoconductive antennas, which was driven by a femtosecond fiber laser to generate and detect terahertz pulses. The femtosecond fiber laser delivered 120 mW, 63 fs de-chirped pulses (after 3 m polarization-maintaining fiber) at a center wavelength of 1560 nm with a repetition rate of 100 MHz. The THz time-domain signal centered at 0.32 THz, ranged from 0.1 to 2.0 THz with the maximum signal-to-noise ratio of 2000:1 was detected and recorded by a Lock-in amplifier.

The samples were placed on a quartz window with a thickness of 3 mm, and then the quartz window was mounted on a manual x-y translation stage. The diameter at the focus point of THz pulses beam was about 2 mm, and the incident terahertz pulses are p-polarized in our system. As shown in Fig. 1a, the reflected THz pulses are composed of two pulses: the first pulse from the air/quartz interface as probe pulse *E*_*prob*_ and the second pulse form the quartz/air or quartz/sample interface as reference pulse *E*_ref_ or sample pulse *E*_sam_, respectively. The *E*_*prob*_ was used to monitor the fluctuation of the THz signals during measurement and calibrate *E*_ref_ and *E*_sam_ according to the fluctuation in complex refractive index calculation. To improve the accuracy of the calculation furthermore, we measured the baseline signal with a thicker (5 mm) quartz window. All the measurements were performed under room temperature at 23 °C with ~50% humidity.

### Data analysis

The complex refractive index of a sample was acquired by the following equations through Fresnel’s coefficient in a reflection mode and Snell’s law^40^

1 $$\frac{\tilde{{n}_{s}}}{\cos \,{\theta }_{3}}=\frac{\tilde{{n}_{s}}\tilde{{n}_{q}}\,\cos \,{\theta }_{2}(R+1)-(R-1){\tilde{{n}_{q}}}^{2}\,\cos \,{\theta }_{1}}{\tilde{{n}_{a}}\,\cos \,{\theta }_{2}(R-1)+\tilde{{n}_{q}}\,\cos \,{\theta }_{1}\,\cos \,{\theta }_{2}(R+1)}$$

2 $${n}_{a}\,\sin \,{\theta }_{1}={n}_{q}\,\sin \,{\theta }_{2}={n}_{s}\,\sin \,{\theta }_{3}$$

where $\tilde{{n}_{a}}$, $\tilde{{n}_{q}}$, $\tilde{{n}_{s}}$ are the complex refractive indices, and *n*_*a*_, *n*_*q*_, *n*_*s*_ are the real part of the complex refractive indices in air, quartz and the sample, respectively. The incident angle of THz pulses is *θ*_1_, and the angles of refraction in the quartz and the sample are *θ*_2_ and *θ*_3_ as shown in Fig. 1a. *R* could be attained from experiment data, $R=\frac{{E}_{{\rm{sam}}}}{{E}_{{\rm{ref}}}}$. We acquired the complex index as a feature value into a database, and a boundary was determined by linear discriminant analysis in a plot of refractive index and absorption coefficient^13^.

In the PCA, the total variation between samples could be expressed in the data set in only a few PCs. And for that all the PCs are orthogonal, the relationships between the different variables is viewed to verify the potential capability of differentiating the *Rheb1* KO and control samples by using terahertz spectroscopy furthermore.

### Statistical analysis

Data represent the mean and SD. Student’s t test (two-tailed) was performed for all statistical significance analysis using GraphPad Prism software. *p < 0.05, **p < 0.01, and ***p < 0.001.
